# Poly beta amino ester coated emulsions of NSAIDs for cartilage treatment†

**DOI:** 10.1039/d1tb01024g

**Published:** 2021-06-25

**Authors:** Tahani Saeedi, Polina Prokopovich

**Affiliations:** Cardiff School of Pharmacy and Pharmaceutical Sciences, Cardiff University Redwood Building King Edward VII Avenue Cardiff CF10 3NB UK prokopovichp@cf.ac.uk

## Abstract

Delivering drugs directly into cartilage is still the major challenge in the management and treatment of osteoarthritis (OA) resulting from the aneural, avascular and alymphatic nature of an articular cartilage structure. Progress has been made in the design of drug delivery systems that enhance corticosteroid uptake and retention in cartilage; however also non-steroidal anti-inflammatory drugs (NSAIDs) are prescribed for patients affected by OA and a drug delivery system specifically designed for this drug category is currently unavailable. We developed an approach based on the preparation of NSAID oil-in-water emulsions coated with poly-beta-amino-esters (PBAEs) to exploit the cartilage penetrating ability of such polymers and the high solubility of drugs in oil. These emulsions containing different NSAIDs (indomethacin, ketorolac, diclofenac and naproxen) exhibited enhanced and prolonged drug localisation not only in healthy cartilage tissues but also in early-stage OA samples. The critical role of the PBAE layer on oil droplets was established along with the retained biological activity of the drug as glycosaminoglycan (GAG) and collagen degradation induced by interleukin-1 (IL-1) was prevented by the novel technology. Oil-in-water coated emulsions are very flexible and cost-effective drug delivery systems and such an approach presented here could provide a substantial improvement in the therapeutic treatments of OA and thus patients’ outcomes.

## Abstract 2

Delivering drugs directly into cartilage is still the major challenge in the management and treatment of osteoarthritis (OA) resulting from the aneural, avascular and alymphatic nature of an articular cartilage structure.

## Graphical abstract

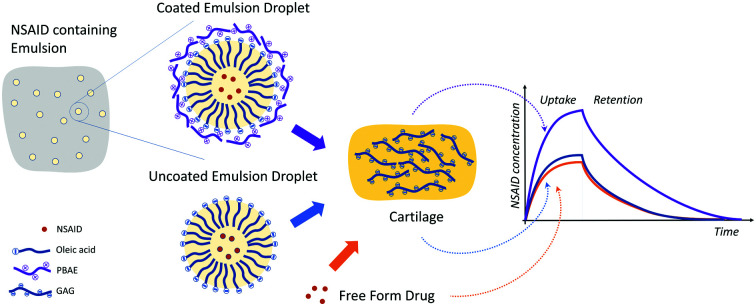

## 1 Introduction

Osteoarthritis (OA) is one of the most prevalent musculoskeletal chronic health conditions; it is characterised by a gradual loss of articular cartilage resulting in joint movement difficulty, stiffness and moderate to severe pain.^1,2^ OA is considered as a “wear and tear” disease because of the strong association between age and incidence.^3–5^ It could affect knees, hands, hip, foot, fingers and many other joints.^4,6,7^ Currently, there is no treatment, but only management to relieve symptoms, improve physical functions as well as the quality of life of patients and prevent arthritis complications and progression.^6,8,9^ The main challenge faced by drugs utilised against OA is their lack of localization, adequate uptake and retention in the tissue;^10^ this is mainly the result of both the no-vasculature nature of joint tissue and the highly electrostatic repulsion towards drugs exhibited by one of the cartilage main constituents (aggrecan);^3^ both factors contribute to quick drug wash-out from the joint requiring treatment.^11^ One of the main approaches to overcome this obstacle is drug encapsulation into micro- or nano-particles for the slow release of drugs into the synovial fluid.^10,12,13^ However, as this method only provides a drug reservoir, it does not encourage penetration of drugs inside the dense extracellular matrix (ECM) of cartilage except when a high drug concentration is used.^14^ Therefore, drug carriers which are specifically designed to promote drug localisation into cartilage, and enhance drug retention in the tissue, are to be favoured to achieve sustained intra-tissue therapeutic levels before they are cleared from the joint space.^10,14–16^

**Fig. 1:**
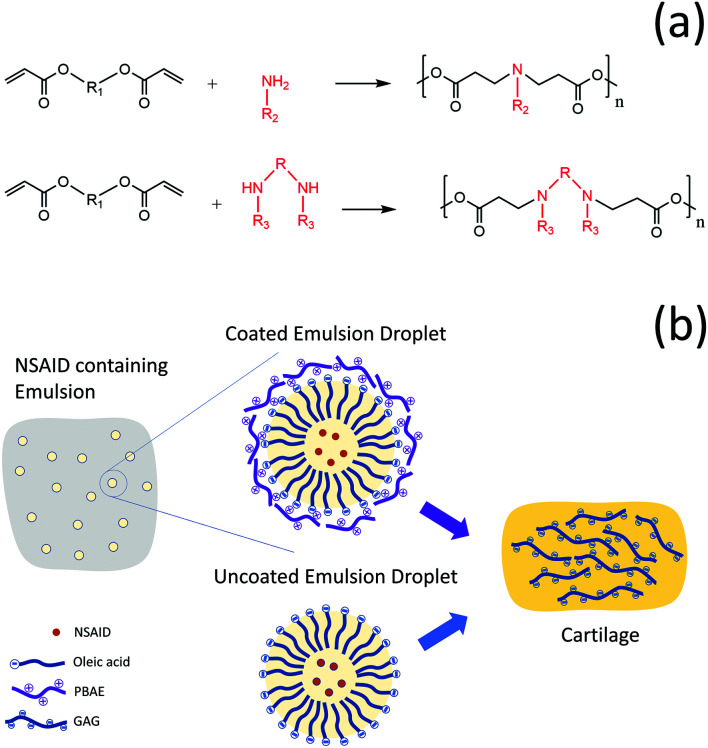
Schematic drawing of PBAE (a) and graphical description of the study approach/design (b).

Poly-beta-amino esters (PBAEs) are positively charged polymers which have shown promising results as drug delivery carriers for corticosteroids in OA treatment.^15,17^ They are synthesised by the co-polymerization of diacrylate and amine monomers^18–20^ and are biodegradable and biocompatible (Fig. 1a). The mechanism of action of PBAE based drug delivery systems for cartilage relies on their electrostatic interaction with the negatively charged aggrecan constituent of cartilage which maximizes transport, uptake, retention and binding of cationic drug carriers inside the tissue.^15,17^

The application of PBAE drug delivery vehicles for cartilage has been, so far, focused on corticosteroids that, despite the widespread application in the management of OA, could also induce potential side effects.^21^ As other therapeutic agents, such nonsteroidal anti-inflammatory drugs (NSAIDs), are also employed in the management of OA,^22^ there is a clear medical need for the improvement of the localisation of this class of molecules in cartilage through the development of a specific drug delivery system.

Coated emulsions are a type of drug delivery system where the active molecule in dissolved in the oil phase of an emulsion and subsequently the oil droplets are coated with a polyelectrolyte that acts as a targeting agent. We hypothesised that PBAEs could be used to deliver drugs into cartilage not only through conjugation of the active compound to the polymer chain but also as the coating agent of emulsions. This would be possible in light of the hydrophobic nature of many drugs that allows their solubilisation in oil and the negative charge of oil drops, when fatty acids are employed, which would enable their coating with the positively charged PBAE (Fig. 1b).

The objectives of this study were to first develop and demonstrate the efficacy of an emulsion system which encapsulates a variety of NSAIDs (indomethacin, ketorolac, naproxen and diclofenac sodium) in an oleic acid emulsion coated with PBAEs to achieve enhanced drug uptake and retention in both healthy and glycosaminoglycan (GAG)-depleted cartilage (mimicking the early stage of OA). The ability of such coated emulsions to inhibit cytokine-induced ECM degradation, rescue cell biosynthesis rates and prevent cell viability loss has been established in an *ex vivo* model of OA.

## 2 Experimental

### 2.1 Chemicals

All amine and acrylate compounds for the synthesis of PBAEs (1,4-butanediol diacrylate and dimethylamino-1-propylamine), sodium acetate, Na_2_HPO_4_ and NaH_2_PO_4_, indomethacin, ketorolac, naproxen, and diclofenac were purchased from Sigma, UK.

Solvents for the polymer synthesis and HPLC mobile phase (dichloro-methane, diethyl-ether, acetonitrile, and acetic acid glacial), and PBS were purchased from Fisher, UK.

All chemicals were used as received and stored as recommended by the manufacturer.

### 2.2 PBAE synthesis

A specific polymer belonging to the PBAE family was used throughout. Typically, 4 mmol of 1,4-butanediol diacrylate were mixed with 4.4 mmol of dimethylamino-1-propylamine in 5 mL of dichloromethane (DCM). The polymerization reaction was then performed under stirring at 50 °C for 48 hours.^15,17,23^ Then, approximately 50 mL of diethyl ether were added to the reaction mixture at room temperature in order to recover the PBAE through precipitation; lastly the solvent was removed under vacuum.^15,17,23^

### 2.3 Emulsion preparation

NSAIDs were dissolved in oleic acid (Thorton & Ross, UK) at a concentration of 40 mg mL^−1^ under magnetic stirring (300 rpm). Then 100 μL of the oil drug solution were added to 900 μL of PBS to obtain 10% o/w emulsion of NSAIDs (final NSAID concentration of 4 mg mL^−1^) and stirred (250 rpm) using a magnetic mixer at room temperature until a stable milky consistency was established (about 24 h).

Emulsions were coated using the saturation method.^24,25^ The specific amount of PBAE to be added to the emulsion was determined, for each specific drug emulsion separately, through zeta potential measurements.

PBAEs were dissolved in 100 mM acetate buffer of pH 5 at a concentration of 2 mg mL^−1^; zeta potentials of PBAE and the emulsion were measured separately. PBAEs were added to 1 mL of the emulsion in 10 μL aliquots, and after each step the zeta potential of the emulsion was measured; saturation was determined when the plateau in the potential was reached.

### 2.4 Zeta potential and hydrodynamic size

The hydrodynamic size (Dynamic Light Scattering (DLS)) and zeta potential of PBAEs and emulsions were measured using a Malvern Zetasizer Nano ZS (Malvern Instruments, Malvern, UK); zeta potentials were calculated from electrophoretic mobility using the Smoluchowski model.

Each data value was an average of three measurements on three independent batches of emulsions.

### 2.5 Cartilage tissue samples

The articular cartilage samples were obtained from 6 to 8 days old immature bovine steer feet which were purchased from the local abattoir. Cartilage explants were surgically extracted from the metacarpo-phalangeal joints of bovine steer feet under sterile conditions. Full depth explants were excised using a 5 mm diameter biopsy puncher from the medial aspect of the medial condyle of the individual joints.^15,17^

GAG-depleted samples were prepared by digesting the cartilage in 500 μL of a PBS solution of trypsin (1 mg mL^−1^) for 24 h at 37 °C followed by washing three times with fresh PBS.^15,17^

Trypsin is a proteolytic enzyme and reduces GAG present in cartilage simulating the early OA stage.^26–28^ The enzyme is dissolved in either PBS^27^ or Na_3_PO_4_^26^ and GAG depletion outcomes appear independent of the aqueous solution.^29^

### 2.6 Drug uptake/retention into cartilage

Cartilage discs (5 mm diameter and approximately 0.5 mm thick) were weighted and placed into the wells of the 96 well plate with the superficial cartilage side facing up. The wells were filled with 100 μL of PBAE coated o/w emulsions containing NSAIDs and placed inside an incubator at 37 °C. Stagnant layers at cartilage surfaces were prevented by placing the plate on a slow-speed rocker. At required intervals (0.5, 1, 2, 3, 5, 7 and 10 min), cartilage samples were removed, washed in plentiful amounts of water and placed in an Eppendorf tube containing 1 mL of digestion buffer.

Comparison of the drug uptake achieved by the PBAE coated-NSAID o/w emulsion (denoted as “coated emulsion”) was performed against 100 μL of a solution in PBS or an uncoated emulsion of the same drug, denoted “drug in PBS” control and “emulsion control” respectively (both controls containing 4 mg mL^−1^ of NSAID).

Drug retention was determined in samples exposed to NSAID containing solutions for 10 min as described above; cartilage samples were washed with an extensive amount of water and placed in an Eppendorf tube containing 0.5 mL of fresh PBS. The cartilage samples were stored at 37 °C for up to 3 h. Samples were removed from the PBS solution at selected time points, washed with water and placed in the Eppendorf tube with 1 mL digestion buffer.

All experiments were performed on both the normal cartilage (healthy condition) and the GAG depleted cartilage (mimicking the early stage of OA). Determinations were performed on triplicate samples originating from 3 different bovine animals using three independent batches of emulsions.

### 2.7 Cartilage digestion

A phosphate buffer (0.2 M pH = 6.8) containing 300 mg L^−1^ of papain, 1 mM EDTA and 2 mM dithiothreitol (DTT) was used to digest the cartilage. Samples were placed in 1 mL of the digestion buffer and incubated at 55 °C for 24 h.^15^

### 2.8 Drug quantification

Reverse phase-HPLC was used to quantify the amount of the NSAID in the digestion buffer. An Agilent series 1100 HPLC system was used with a μBondapak® C18 10 μm 125A analytical column (for indomethacin only) or a Teknokroma TRACE EXCEL 120 ODSB 5 μm (for the other NSAID) maintained at 25 °C.

The injection volume was 20 μL, the mobile phase was pumped at 1 mL min^−1^ and the other operating parameters used were dependent on the NSAID analysed and are shown in Table S1 (ESI†). The amount of drug present in the cartilage was then expressed as the mass of drug per cartilage mass.

### 2.9 Emulsion diffusion in cartilage

Nile red was dissolved in oleic acid (2 μg mL^−1^) and emulsions were prepared as described previously.

Cartilage samples were placed in a 96 well plate with 100 μL of DAPI solution (10 μg mL^−1^ in PBS); the samples were stored for 10 min at room temperature in the dark before washing with PBS three times. The samples were placed in another 96 well plate with 100 μL of emulsion with Nile red (0.2 μg mL^−1^) and incubated for 1 min; after washing with PBS, the samples were placed on a glass slide and covered with a cover slip. Images were acquired using a confocal Zeiss LSM880 microscope with 20× lenses. 40 slices were taken and equally distributed from the superficial section to a depth of 130 μm.

### 2.10 *Ex vivo* model for post-traumatic OA

Cartilage disks were extracted as described before and weighted to normalise the results. The disks were equilibrated in serum-free medium (low-glucose DMEM, l-glutamine, 25 mM HEPES, 110 mg L^−1^ sodium pyruvate), supplemented with 1% insulin-transferrin selenium (corresponding to insulin 10 μg mL^−1^, transferrin 5.5 μg mL^−1^ and selenium 5 ng mL^−1^), 0.1 mM nonessential amino acids (Gibco, UK), 4M proline (Sigma Aldrich, UK), 20 mg mL^−1^ ascorbic acid (Fisher Scientific, UK), 100 units per mL penicillin G, 100 μg mL^−1^ streptomycin and 250 μg mL^−1^ amphotericin B (Sigma Aldrich, UK) for two days prior to treatment at 37 °C in 5% CO_2_. Cartilage explants were treated for 14 days with IL-1α (1 ng mL^−1^) to induce GAG loss.^17,30^

Samples (*n* = 6) were incubated at 37 °C in a humidified atmosphere containing 5% CO_2_; the medium was changed every 2 days. The following treatments were compared: pure medium, medium containing IL-1α, medium containing IL-1α and 4 mg mL^−1^ ketorolac, which was delivered as ketorolac solution in PBS, ketorolac emulsion or ketorolac containing emulsion coated with PBAE. A “continuous dose” of ketorolac was maintained for 20 days by replenishing it at the same time as the medium; while for the “single dose” experiment, ketorolac was in the culture medium only for the first 2 days. IL-1α was replenished at each medium change for the sample.^17^

#### 2.10.1 Quantification of GAG and collagen in cartilage

Glycosaminoglycan content in cartilage explants was measured using dimethyl-methylene blue (DMMB) dye assay.^31^

Collagen content was measured using the hydroxyproline assay.^32^

#### 2.10.2 Live/dead cell staining of cartilage

Plasma membrane integrity of the chondrocytes in the cartilage was visualised using a Live/Dead cell staining kit (Invitrogen, UK) which utilises calcein-AM (calcein acetoxymethyl ester) and ethidium homodimer-1. The live/dead staining solution (1 μM calcein-AM and 1 μM ethidium homodimer-1 in PBS) was prepared by adding 5 μL of calcein-AM and 20 μL of ethidium homodimer-1 to 10 mL sterile PBS and used immediately.

At set intervals, the samples were removed from the medium and 100–150 μm thick cross-sectional slices were cut from the centre of the cartilage disks exposed to each type of treatment using stainless steel single-use sterile surgical scalpels (Fisher, UK).^33^ The cartilage slices were placed in a 96 well plate and directly stained with 200 μL of the prepared live/dead staining solution for 30 minutes in the dark at room temperature. Then, the tissue slices were washed 3 times with PBS and imaged using a 10× objective lens on the confocal microscope LSM (Zeiss LSM880).

The viability of chondrocytes in the cartilage explants was assessed as calcein AM (excitation/emission ∼493–582 nm) stained viable cells in green and ethidium homodimer-1 (excitation/emission ∼582–741 nm) stained non-viable cells in red.

#### 2.10.3 Histological analysis of cartilage tissue

Cartilage tissue was examined histologically on day 20 of the incubation period to visualize and quantify proteoglycan (GAG) content. The cartilage tissues were fixed in 10% (v/v) neutral buffered formalin (Fisher, UK) for 48 h and dehydrated through increasing the concentration of ethanol followed by xylene.

Cartilage samples were then processed for paraffin embedding and histology with 5 μm thick serial sections using a microtome. Sections were de-paraffinized and stained with safranin-O (Fisher, UK) using the standard procedure.^34^ The tissue sections were dehydrated and mounted. Images were captured using a light fluorescence microscope (Leica DMRB) controlled by Zen Pro software 2012.

A quantitative analysis of staining intensity was performed extracting from the images the colour brightness of the RGB channels on 20 random sections perpendicular to the sample surface using R.^35^

#### 2.10.4 Tissue viability assay

Cartilage tissue viability was analysed using in an XTT based on an *in vitro* toxicology assay kit (Sigma-Aldrich, UK) with a small adaptation to the manufacturer's protocol. In brief, pre-weighed cartilage tissues were cultured in media for 20 days. Then, they were cut using a scalpel into approximately 4–6 pieces and incubated in the XTT solution (1 mL) for 4 h at 37 °C in 5% (v/v) CO_2_ in air. Then, the XTT solution was removed and retained to be used later. After that, 0.5 mL of dimethyl sulfoxide (DMSO) were added and incubated for 1 h to extract the tetrazolium product from the tissue. XTT and DMSO solutions were then mixed before reading the absorbance of triplicate samples at 450 nm and 690 nm respectively in a 96 well plate using a spectrophotometer (Tecan, Infinite 200 PRO). Absorbances at 690 nm were subtracted from the absorbances measured at 450 nm and the XTT content was calculated per gram of cartilage tissue.

### 2.11 Statistical analysis

Statistically significant differences between the mean of NSAID uptake and retention in each type of control were tested using a paired sample *t*-test or one-way ANOVA with a Tukey's *post hoc* test (*p* < 0.05). Differences in GAG, collagen content and XTT assay were analysed using one-way ANOVA followed by Tukey's *post hoc* test (*p* < 0.05).

The statistical analysis was performed using IBM SPSS Statistics (Version 25).

## 3 Results

### 3.1 Emulsions

#### 3.1.1 Zeta potential measurements

PBAE exhibited a positive charge (+25.2 ± 0.9 mV [mean ± SD, *n* = 3]) while the oil droplets without any NSAID had a negative charge (−41.1 ± 2.5 mV [mean ± SD, *n* = 3]). The presence of indomethacin and naproxen had little impact on the zeta potential of the emulsion (−45.8 ± 8.5 and −46.6 ± 1.9 mV [mean ± SD, *n* = 3], respectively); on the other hand, ketorolac and diclofenac resulted in an oil droplet with a greater negative potential (−73.9 ± 0.5 and −84.6 ± 3.4 mV [mean ± SD, *n* = 3], respectively).

**Fig. 2:**
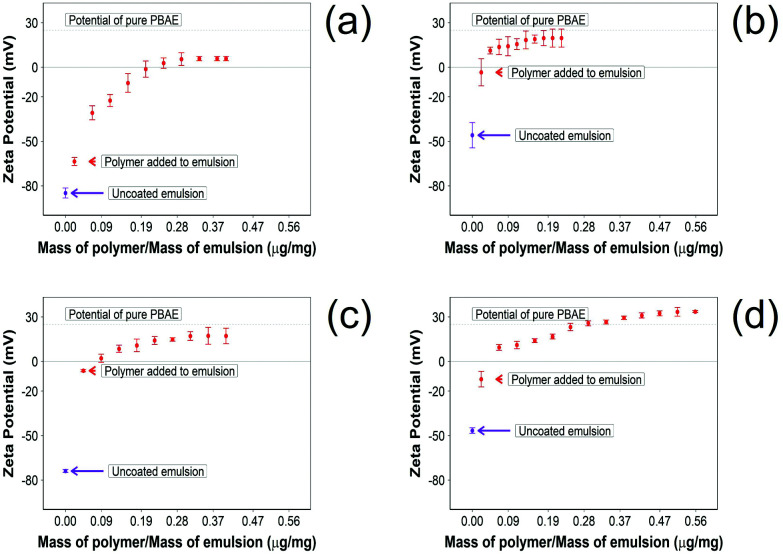
Zeta potential measurements 10% w/o NSAID emulsions during PBAE coating. (a) Diclofenac, (b) indomethacin, (c) ketorolac and (d) naproxen (mean ± SD, *n* = 3).

The addition of PBAE to the emulsions progressively changed the zeta potential from a negative value to positive values until complete saturation (Fig. 2). For indomethacin and ketorolac containing emulsions, the saturation was achieved after addition of 0.18 μg of PBAE every mg of emulsion, while for naproxen 0.52 μg mg^−1^ was required. In the case of diclofenac, 0.30 μg mg^−1^ of PBAE was needed to fully coat the emulsion droplets.

In addition, after coating the NSAID o/w emulsion with PBAE, naproxen showed a higher positive charge compared with other drug containing emulsions; emulsions containing indomethacin and ketorolac reached the potential of pure PBAE once coated, while droplets with diclofenac did not reach the potential of pure PBAE.

Pure oil emulsions required 0.13 μg of PBAE per mg of emulsion to reach full saturation of the charges.

#### 3.1.2 Size measurement

**Fig. 3:**
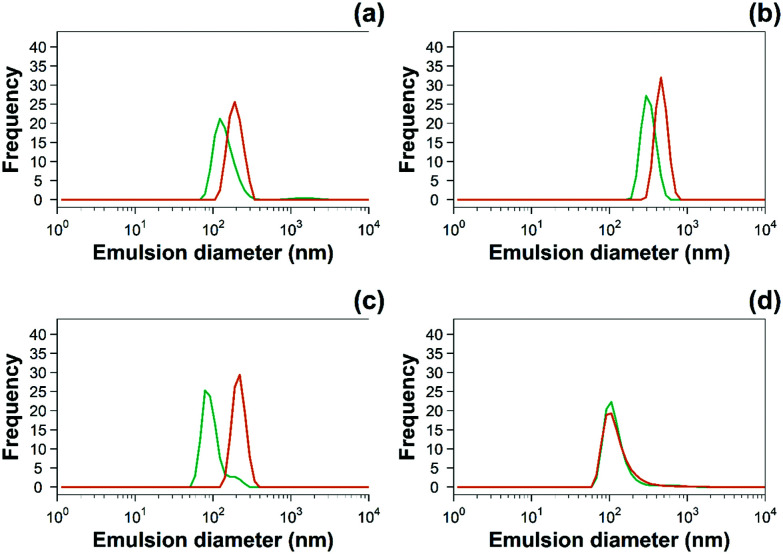
Examples of hydrodynamic size distribution of 10% w/o (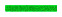) uncoated and (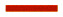) PBAE coated emulsions of (a) diclofenac, (b) indomethacin, (c) ketorolac and (d) naproxen.

Emulsion sizes were normally distributed (Fig. 3) regardless of the PBAE coating or the drug added; uncoated emulsions of NSAIDs had oil droplets with similar diameters of ∼150 nm; pure emulsions of oleic acid in water had a larger diameter of 660 nm (Table 1); the size of emulsion droplets increased after being coated with PBAE. For example, diclofenac containing emulsion droplet size increased approximately ten times from 120 ± 30 nm to 190 ± 35 nm after the coating while emulsion droplets with indomethacin had double diameter after PBAE coating. Overall, the indomethacin coated emulsion exhibited the largest droplet size and the naproxen containing emulsion had the smallest droplet size compared to the formulations with other NSAIDs (Table 1).

**Table 1:** Size measurements for pure 10% o/w and NSAIDs containing emulsions (mean ± SE, *n* = 3)

Emulsion	Size (nm) – uncoated	Size (nm) – PBAE coated
Emulsion only (o/w)	660 ± 15	880 ± 130
Indomethacin	200 ± 20	460 ± 330
Ketorolac	80 ± 10	220 ± 30
Naproxen	110 ± 25	110 ± 30
Diclofenac sodium	120 ± 30	190 ± 35

### 3.2 Drug uptake into cartilage

**Fig. 4:**
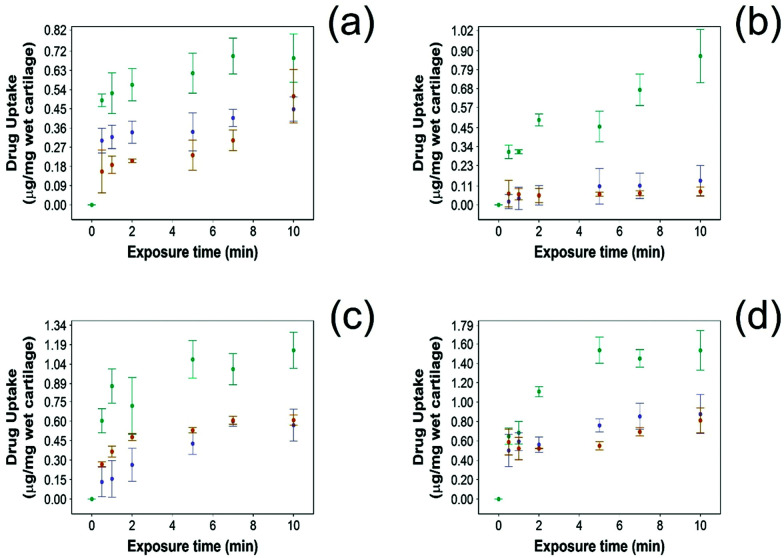
Comparison of NSAID uptake into cartilage using 10% w/o PBAE coated emulsions (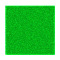), drug in PBS (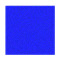) and 10% w/o emulsion controls (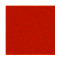) in healthy cartilage. (a) Diclofenac, (b) indomethacin, (c) ketorolac and (d) naproxen (mean ± SD, *n* = 3).

For all NSAIDs encapsulated in the coated emulsions, drug uptake steadily increased with exposure time until it reaches a saturation at 10 min (Fig. 4). At each time point, a statistically significant higher amount of drug (*p* < 0.05) was measured inside the cartilage tissue when coated emulsions were employed compared to “drug in PBS” and “emulsion” controls (Fig. 4).

**Fig. 5:**
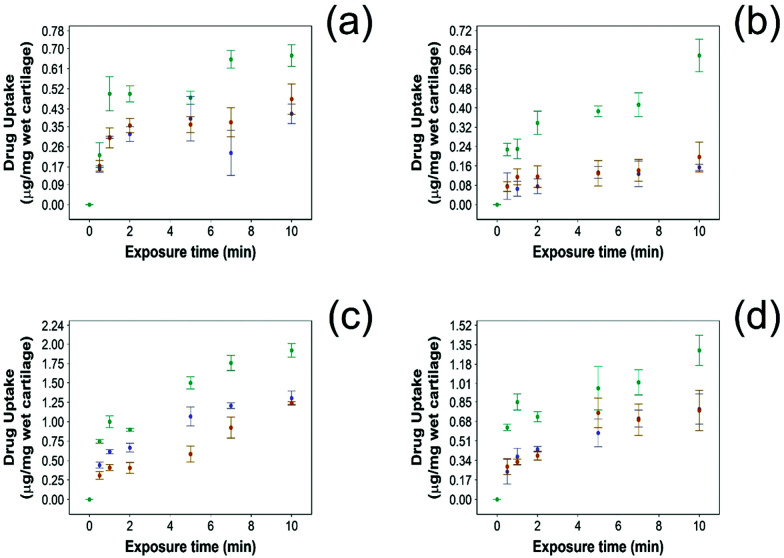
Comparison of NSAID uptake into cartilage using 10% w/o PBAE coated emulsions (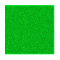), drug in PBS (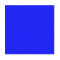) and 10% w/o emulsion controls (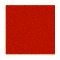) in GAG-depleted cartilage. (a) Diclofenac, (b) indomethacin, (c) ketorolac and (d) naproxen (mean ± SD, *n* = 3).

Additionally, NSAID uptake by cartilage was higher in healthy cartilage than in GAG depleted cartilage (*p* < 0.05) (Fig. 5) as the healthy cartilage has a significantly higher amount of GAGs allowing stronger electrostatic interactions between the positively charged PBAE coating of the emulsion and the negatively charged GAGs. However, despite relying on the electrostatic attraction between the positive charges of the PBAE coating of the emulsion and negative charges of the GAG molecules to deliver the drug into the cartilage tissue, the developed coated emulsion system was nevertheless effective on GAG depleted cartilage (Fig. 5) as the amount of drug detected in the cartilage tissue after exposure of an equal concentration of NSAID was higher in the case of the coated emulsion than “drug in PBS” and “emulsion” control (*p* < 0.05).

### 3.3 Drug retention into cartilage

**Fig. 6:**
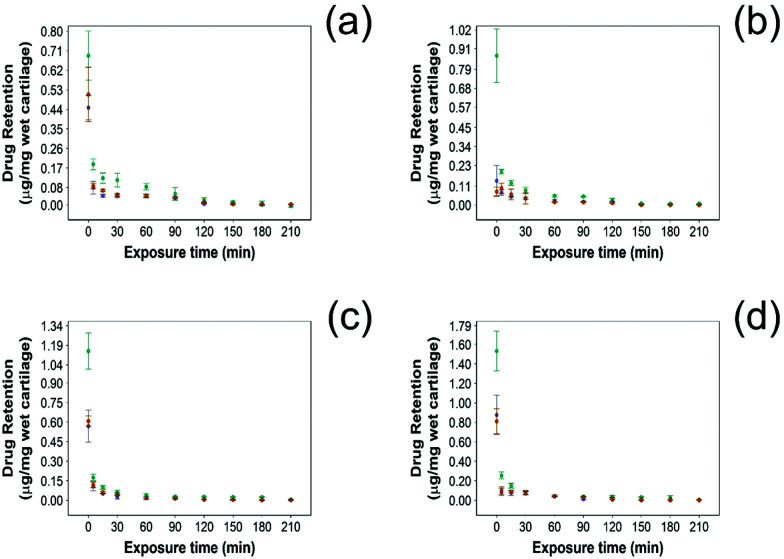
Comparison of NSAID retention into cartilage using 10% w/o PBAE coated emulsions (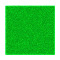), drug in PBS (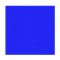) and 10% w/o emulsion controls (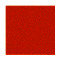) in healthy cartilage. (a) Diclofenac, (b) indomethacin, (c) ketorolac and (d) naproxen (mean ± SD, *n* = 3).

**Fig. 7:**
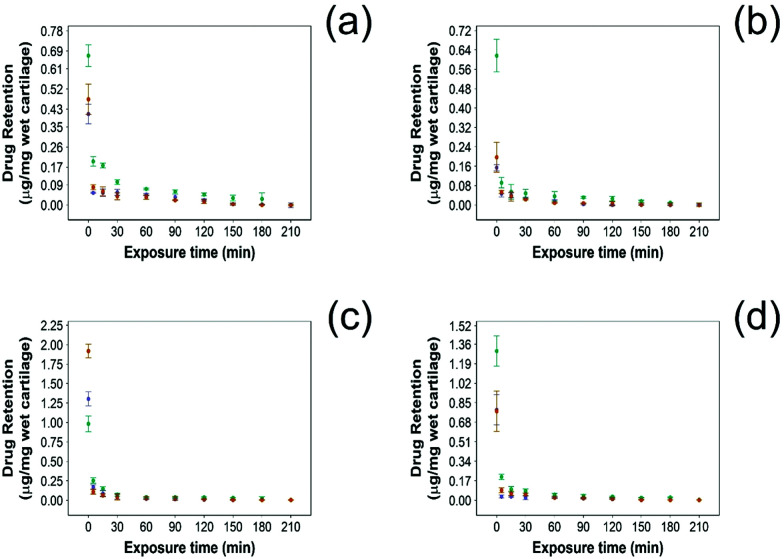
Comparison of NSAID retention into cartilage using 10% w/o PBAE coated emulsions (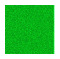), drug in PBS (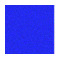) and 10% w/o emulsion controls (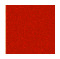) in GAG-depleted cartilage. (a) Diclofenac, (b) indomethacin, (c) ketorolac and (d) naproxen (mean ± SD, *n* = 3).

In this study NSAID retention in cartilage was better for the developed emulsion system compared to other systems reported, although the majority of the drug was released from the cartilage in the first 60 min post uptake (Fig. 6 and 7). In healthy cartilage the drug concentration fell below the detection limit after 1.5–2 h in the case of “drug in PBS” and “emulsion” control when the coated emulsion was used NSAID concentration was still detectable in the cartilage tissue even after 3 h (Fig. 6). In GAG depleted cartilage (Fig. 7) drug release was faster than in healthy cartilage tissues, as the drug was not detected after 2 h in all cases.

Additionally, the amount of NSAID remaining in the tissue was always higher when delivered through PBAE coated emulsions than for “drug in PBS” and “emulsion” control (*p* < 0.05) in both healthy and GAG depleted cartilage tissues.

### 3.4 Emulsion diffusion through cartilage

**Fig. 8:**
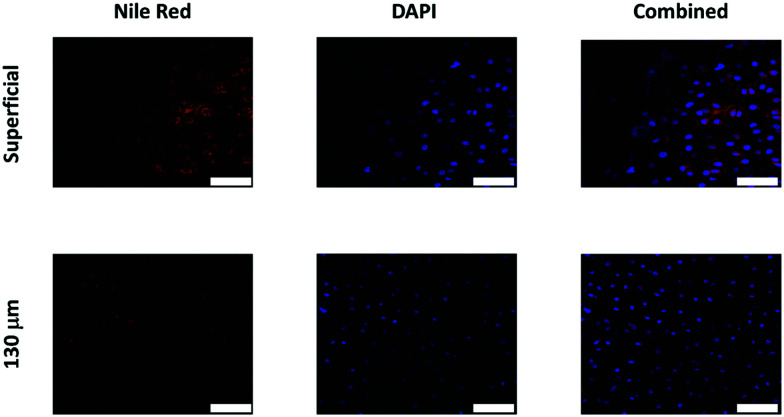
Examples of confocal images of Nile red fluorescently tagged oil emulsion diffusion through cartilage with chondrocytes countered stained with DAPI. Scale bar = 50 μm.

Oil droplets were detectable inside chondrocyte cells as both DAPI (tagging cells) and Nile red (tagging oil droplets) emissions were overlapping in the images observed. Moreover, Nile red containing oil droplets were detected not only on the surface of the cartilage samples but also at least 130 μm deep (Fig. 8).

### 3.5 *Ex vivo* model for post-traumatic OA

#### 3.5.1 GAG loss measurement

**Fig. 9:**
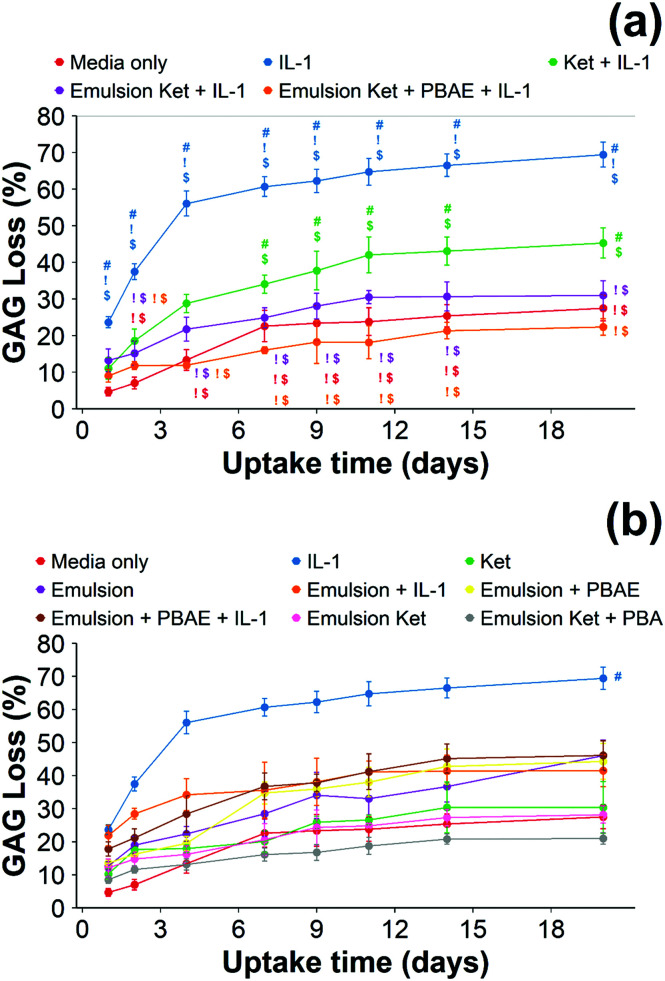
Quantification of GAG using DMMB assay in IL-1α (1 ng mL^−1^) treated cartilage. Main samples (a) and controls (b) separated for clarity (mean ± SD, *n* = 6) (# *vs.* media only, ! *vs.* ketorolac + IL-1α, $ *vs.* continuous dose Ket through coated emulsions *p* < 0.05. Statistical markers are colour coordinated with the curves. All data enclosed within statistical markers are significant.)

GAG loss from cartilage tissues treated with IL-1α alone was faster during the first week of incubation (Fig. 9); after cumulative GAG loss from IL-1 α treated cartilage was ∼60% and significantly higher than from the control samples (*p* < 0.01). Overall, the percentage of GAG loss was 3–5 times higher in IL-1α treated cartilage than in the untreated group (in media only). Ketorolac was able to reduce GAG loss to ∼40% after 20 days of culture.

When cartilage samples were continuously exposed to IL-1α and coated or uncoated emulsions with and without ketorolac a remarkable decrease in GAG loss was observed (*p* < 0.01) (Fig. 9). The loss of GAGs using the coated emulsions was compatible with the untreated control and it was lower than the uncoated emulsions containing ketorolac (*p* < 0.05).

When ketorolac was delivered as single dose, the protective effect of ketorolac on GAG reduction induced by IL-1α was reduced (Fig. 11); but after 20 days of exposure, the difference between the delivery through the coated emulsion and either the uncoated emulsion or pure drug was statistically significant (*p* < 0.01).

#### 3.5.2 Collagen content

**Fig. 10:**
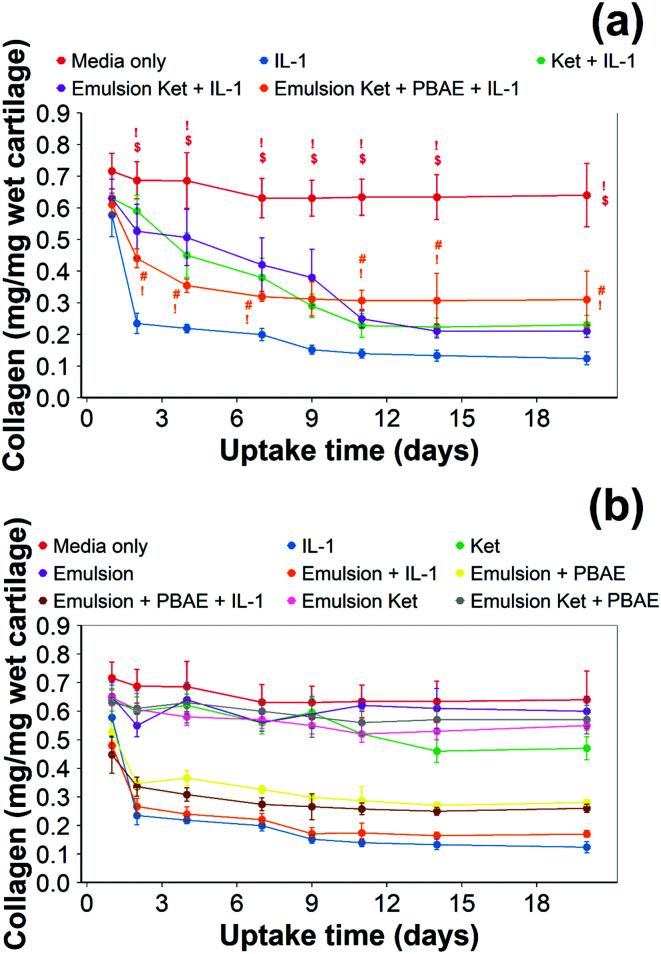
Quantification of collagen using hydroxyproline assay in IL-1α (1 ng mL^−1^) treated cartilage. Main samples (a) and controls (b) separated for clarity (mean ± SD, *n* = 6) (# *vs.* media only, ! *vs.* IL-1α, $ *vs.* continuous dose Ket through coated emulsions *p* < 0.05. Statistical markers are colour coordinated with the curves. All data enclosed within statistical markers are significant.)

Collagen content dropped to half the initial amount during the first week of tissue culture exposed to IL-1α and decreased steadily after that (Fig. 10). Overall, collagen degradation was statistically significant following IL-1α compared to the controls (*p* < 0.05).

Continuous addition of pure ketorolac or the uncoated emulsion of the drug reduced the collagen degradation compared to the IL-1α treated media/control; moreover, the greatest collagen degradation reduction was achieved in PBAE coated emulsions returned the greatest collagen degradation reduction.

**Fig. 11:**
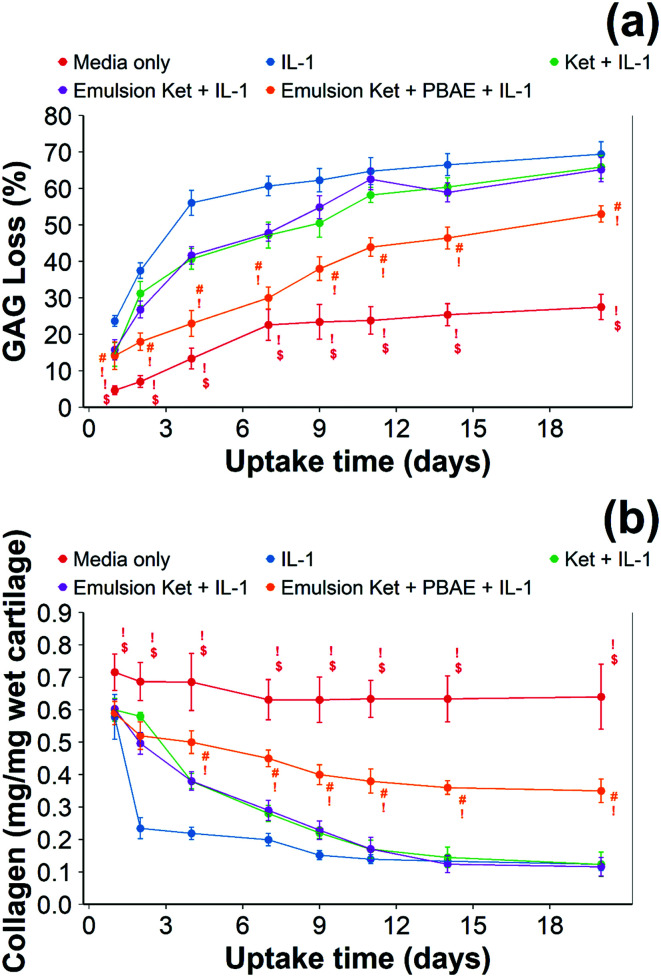
Quantification of GAG using DMMB assay (a) and collagen using hydroxyproline assay (b) in IL-1α (1 ng mL^−1^) treated cartilage (mean ± SD, *n* = 6) (# *vs.* media only, ! *vs.* ketorolac + IL-1α, $ *vs.* single dose Ket through coated emulsions *p* < 0.05. Statistical markers are colour coordinated with the curves. All data enclosed within statistical markers are significant.)

When ketorolac was delivered as single dose, the protective effect of ketorolac on collagen degradation induced by IL-1α was reduced (Fig. 11); but after 20 days of exposure, the difference between the delivery through the coated emulsion and either the uncoated emulsion or pure drug was statistically significant (*p* < 0.01).

#### 3.5.3 Live/dead viability assay

**Fig. 12:**
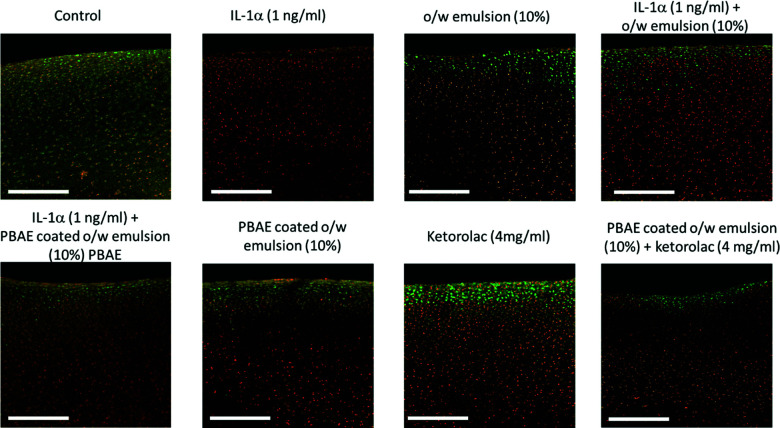
Examples of images of live/dead fluorescently stained bovine explants exposed to IL-1α (1 ng mL^−1^) and ketorolac containing emulsions after incubation for 20 days. Scale bar = 500 μm.

Images of cartilage treated with IL-1α showed decreasing chondrocyte viability compared to the untreated control (Fig. 12). The presence of ketorolac in coated emulsions helped to maintain high cell viability, even in the presence of IL-1α, during the first week of tissue culture alongside. Exposure to the PBAE coated emulsion of ketorolac showed improved chondrocyte viability to levels comparable to the control/untreated group (Fig. 12).

#### 3.5.4 Histological analysis of cartilage tissues

**Fig. 13:**
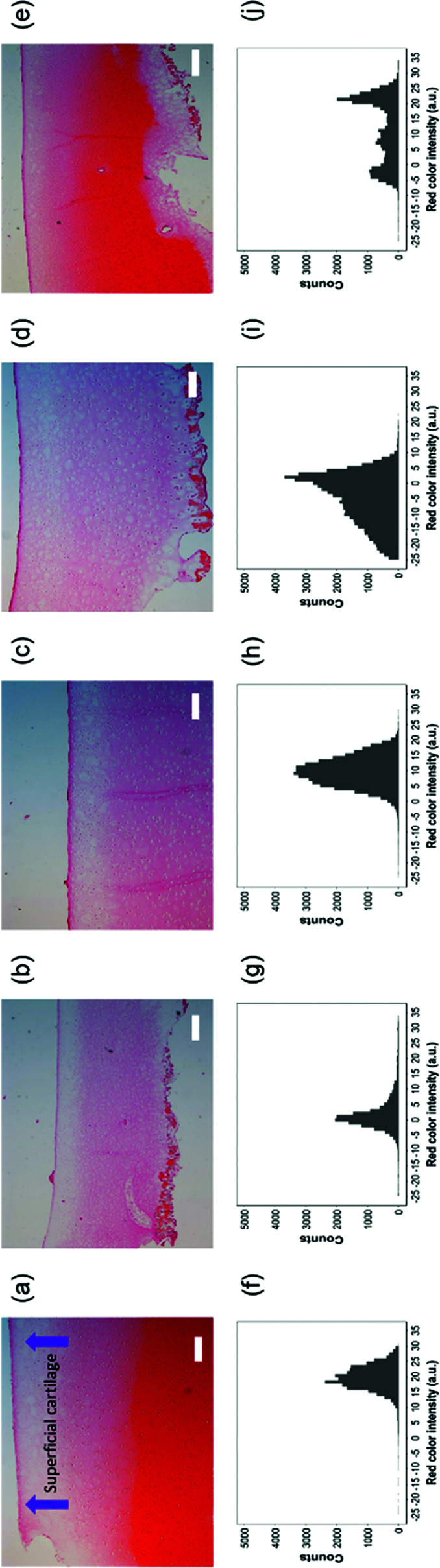
Top row, safranin-O stained histology sections of cartilage exposed to (a) pure media, (b) IL-1α (1 ng mL^−1^), (c) IL-1α (1 ng mL^−1^) + ketorolac (4 mg mL^−1^), (d) IL-1α (1 ng mL^−1^) + ketorolac (4 mg mL^−1^) uncoated emulsion and (e) IL-1α (1 ng mL^−1^) + ketorolac (4 mg mL^−1^) PBAE coated emulsion for 20 days. Scale bar = 200 μm. Bottom row, histograms of red colour intensity for the corresponding samples.

Images of the sections of the cartilage tissues stained with safranin-O are shown in Fig. 13. The results of the control tissue sections stained with safranin-O revealed a high stain intensity of GAGs at day 20. In the cartilage tissue treated with IL-1α, there was a significant reduction in the stain intensity compared to the control sample at day 20 indicating a substantial loss of GAGs from the tissue.

**Fig. 14:**
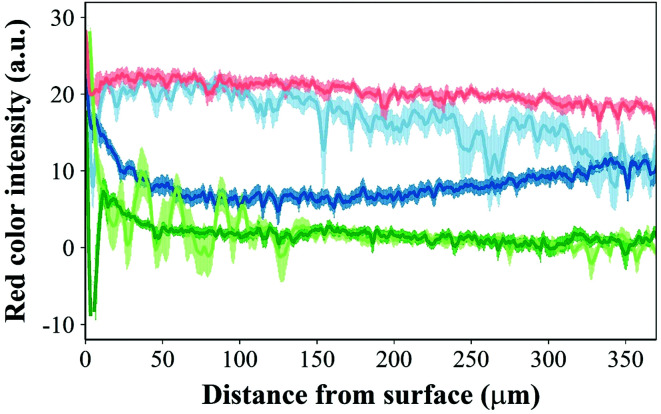
Profile of red intensity in safranin-O stained histology sections for cartilage exposed to (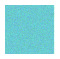) IL-1α (1 ng mL^−1^) + ketorolac (4 mg mL^−1^) PBAE coated emulsion, (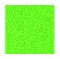) IL-1α (1 ng mL^−1^), (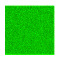) IL-1α (1 ng mL^−1^) + ketorolac (4 mg mL^−1^), (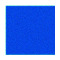) IL-1α (1 ng mL^−1^) + ketorolac (4 mg mL^−1^) uncoated emulsion and (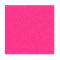) pure media for 20 days (mean ± SD, *n* = 20).

Safranin-O intensity in samples cultured in pure medium was uniform from the cartilage outer layer towards the bulk (Fig. 14). However, there was a drop when tissues were exposed to IL-1α with the highest intensity on the surface of cartilage. Ketorolac in medium containing IL-1α had an impact on the safranin-O intensity profile only when administered through an emulsion; furthermore, PBAE coated emulsions resulted in results similar to those observed for cartilage cultured in pure medium.

#### 3.5.5 Cartilage tissue viability analysis

**Fig. 15:**
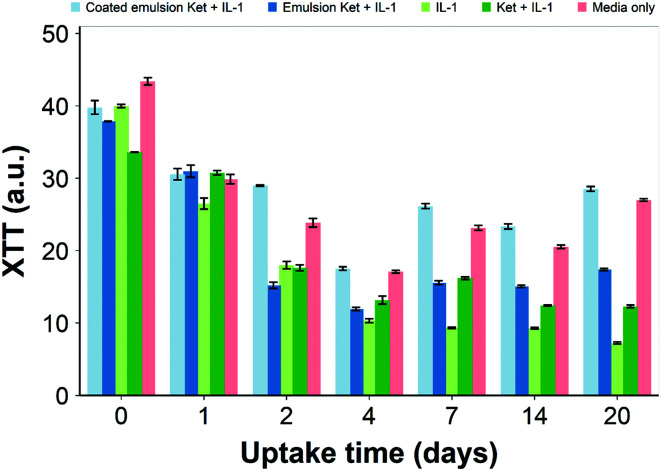
Viability assessment of cartilage tissues over 20 days (mean ± SD, *n* = 6).

XTT assay (Fig. 15) of the cartilage tissue viability indicated that the cartilage tissue showed reduced XTT conversion or mitochondrial activity from day 0 to 20 when exposed to IL-1α compared to the untreated control (*p* < 0.01). Treating the cartilage tissue with IL-1α led to a 4 times decrease in cartilage cell mitochondrial activity during the culture period (20 days). Addition of ketorolac with and without the emulsion led to a slight increase in chondrocyte mitochondrial activity compared to cartilage samples treated with IL-1α alone. However, treating the tissue samples with the PBAE coated emulsions of ketorolac led to a significant increase in the cell mitochondrial activity (*p* < 0.01) reaching a level similar to that of cartilage samples not exposed to IL-1α.

## 4 Discussion

Intra-articular administration of drugs has potential benefits over systemic administration in the treatment of OA, *i.e.* targeting only the joint of interest with reduced side effects; however, the joint cartilage extracellular matrix is characterised by highly negatively charged proteoglycans hindering the ability of small molecule drugs to localise into the articular cartilage and favouring clearance from the affected joints.^10,11^ For example, NSAID half-lives in cartilage/joints have been reported to be 1–4 hours.^11,36^ Nevertheless, the high negative charges of the cartilage matrix also provide a great opportunity to improve and facilitate the uptake and retention of the positively charged drug or drug carriers.^14–16,26^ We have developed a novel drug delivery system for NSAIDs encapsulating them in the oil phase of an emulsion prepared with oleic acid that is fully coated with PBAE. NSAIDs have anti-inflammatory activity but have also been shown to have a positive effect on the cartilage degeneration in OA patients.^37^ This approach is based on the positive charge of PBAE, their confirmed biocompatibility and ability to drive drug uptake in cartilage through electrostatic interactions with the negatively charged proteoglycans.^15,17^ The system was effective in enhancing drug uptake and extending drug retention of numerous NSAIDs clinically used for managing OA. Moreover, IL-1α induced cartilage GAG and collagen were prevented by ketorolac delivered through the novel PBAE coated emulsion droplets, which was also instrumental in retaining chondrocyte viability.

In this work, one representative of the PBAE class was employed to determine the feasibility of the approach; the choice of using the copolymer of 1,4-butanediol diacrylate and dimethylamino-1-propylamine was elicited by the experimentally observed high zeta potential and cartilage penetrating ability of this particular polymer.^17^ Oleic acid was chosen because of its biocompatibility, low cost and ability to form emulsions; furthermore because of the negative charges of the carboxyl groups the oil droplets of emulsions prepared with oleic acid are amenable to coating with PBAE.

The zeta potential and size of oil droplets depended on the drug encapsulated in the oil phase; loading the emulsion with NSAIDs resulted in a more negative zeta potential than for the pure oleic acid emulsion due to the deprotonation of the carboxylic group at the oil/water interface. Diclofenac containing samples showed the more negative value of zeta potential possibly due to the positive charges stabilising the action of secondary nitrogen present in its structure. The surface activity of drugs dissolved in the oil phase adsorbed at the oil/water interface decreased the interfacial tension resulting in smaller droplets for NSAID containing emulsions. The addition of a polyelectrolyte with positive charges (PBAE) progressively saturates the negative charges present on the oil droplet surface with a consequent initial increase in zeta potential and subsequent switch to positive values. At saturation, the zeta potential of the coated oil droplets was similar to that of pure PBAEs in the case of indomethacin and ketorolac (Fig. 2); when the polymer does not fully saturate the original droplet charges the potential of the coated emulsion is lower than the coating polyelectrolyte (diclofenac). Coated emulsions also had larger droplets (Fig. 3) caused by coalescing drops when the zeta potential approached neutrality reducing the emulsion stability caused by electrostatic repulsion or by the aggregation of drops when polyelectrolytes simultaneously interact with more than one drop as already seen in silica nanoparticles coated with PBAE.^38^ Nile red was dissolved in oleic acid to fluorescently tag the emulsions and confirm whether the droplets actually diffuse through the cartilage tissue or just adsorb onto the outer surface of the samples, although the size of the droplet was greater than the ∼10 nm threshold reported previously.^14^ The diffusion of the coated emulsion and other large therapeutic agents can be explained by the fact that, despite the spacing between glycosaminoglycan (GAG) molecules in cartilage being ∼5 nm, molecules/vesicles larger than 5 nm can move between collagen fibrils which exhibit a space of about 50–100 nm in size.^39^ Such dimensions also explain how micelles, liposomes and other micro-scale particles have been successfully developed for intra-articular drug delivery to treat OA.^40–42^

The working mechanism of the drug delivery system presented in this work depends mainly on the electrostatic interaction between the positive charged PBAE and the negatively charged GAG; therefore, a decreased efficacy in the GAG depleted cartilage was expected (Fig. 5). Like these results, avidin uptake was significantly higher in healthy cartilage than the GAG depleted one.^15,26^ As it is expected that real life applications of the proposed technology would predominantly be consisting of GAG depleted tissues, it was essential to ascertain the efficacy of the coated emulsion system in such OA mimicking samples. Although the electrostatic interactions between GAG and PBAE are a key driving force in drug uptake, the porosity of cartilage, offering hydrodynamic resistance to fluid flow through the tissue, also impacts such a phenomenon. Thus, it is also possible that the higher porosity of the GAG depleted cartilage^43,44^ partially counteracts the reduced electrostatic interaction in such samples as they display higher hydraulic permeability than normal matrices allowing better flow.

Drug retention determines, along with drug uptake, the duration of the therapeutic range when the drug concentration is above the minimum required to exert its biological activity. As the same factors affecting drug uptake also control its retention, it was expected that the PBAE coated emulsions would not only enhance drug localisation but also prevent its release. We have shown that the vast majority of NSAIDs were still detected in the cartilage samples even after 3 hours of incubation in PBS for both healthy and GAG depleted cartilage (Fig. 6 and 7) and at concentrations consistently higher than when a drug was administered in its pure form or in uncoated emulsions.

Encapsulating the drug in emulsions to improve localisation in cartilage could hypothetically prevent them from exerting activity towards chondrocytes as the active molecule required to be released from the oil phase. We expected this to be the result of PBAE hydrolysis and metabolization of oleic acid. In order to assess the biological activity of drugs conveyed into cartilage through the proposed delivery system, we employed ketorolac to contrast the proteolysis of the cartilage ECM induced by IL-1α with a protocol similar to other previously employed ones to test the efficacy of dexamethasone delivery systems.^17,30^ OA and traumatic joint injury, which progress to post-traumatic OA, are characterised by increased synovial fluid concentrations of pro-inflammatory cytokines such as IL-1α and TNFα.^45,46^ IL-1α is known to induce catabolic responses in chondrocytes, and it is a potent inducer of prostaglandin (PG) synthesis in human chondrocytes;^47^ moreover, IL-1α causes GAG and collagen loss.^48,49^ Moreover, the chondrocyte viability reduction in samples exposed to IL-1α (Fig. 12) was also observed using the same cytokine concentration.^30^

Ketorolac was chosen as a model NSAID for *ex vivo* tests as it showed a significantly better uptake and retention results compared to the delivery systems with other NSAIDs present. IL-1α caused significant GAG and collagen losses in cartilage compared to samples cultured in pure medium. Furthermore, ketorolac reduced the ECM proteolytic effect of IL-1α and its encapsulation in coated emulsion provided a significant increase (*p* < 0.01) in GAG and collagen content during the culture time to levels similar to the untreated control (Fig. 9 and 10) revealing that the drug is not only localised in the tissue but also the delivery system released the cargo allowing the drug to act. Moreover, treatment cartilage with ketorolac coated emulsions did not exhibit toxicity towards chondrocytes as there was no observed effect on chondrocyte viability using live/dead assay and XTT assay (Fig. 12 and 15). GAG determined through DMMD assay is the average content of the sample; however, GAG is not homogeneously distributed and safranin O staining also allows the investigation of its spatial distribution and thus we were able to determine that the recovery observed with NSAIDs was also restoring the necessary distribution (Fig. 13 and 14).

## 5 Conclusions

Emulsion based drug delivery systems exhibit advantages over the conjugation of drugs to targeting polymers; for example, drug loading is not restricted by the ratio between the polymer length and binding sites while concentration is not constrained by the aqueous solubility of the conjugate. Moreover, preparation of emulsions does not incur losses and thus is a more efficient and cost-effective production route. This work revealed that a NSAID and oleic acid-based o/w emulsion coated with PBAE is an effective delivery system for cartilage due to the electrostatic forces of attraction between the negatively charged GAGs in the tissue and the positively charged PBAE.

The drug delivery system was functional in both healthy and GAG-depleted cartilage (mimicking the early stage of OA); in addition, ketorolac emulsions coated with PBAE were capable of preventing the catabolic effect exerted by IL-1α without impacting chondrocyte viability and metabolic activity.

## Conflicts of interest

PP is a named inventor in the patent application related to the application of PBAE to cartilage treatment. TS has no conflict to declare.

## Supplementary Material

TB-009-D1TB01024G-s001
