# Supplementary material for: Poly beta amino ester coated emulsions of NSAIDs for cartilage treatment
Source: J Mater Chem B. 2021 Jun 25;9(29):5837–47. doi: 10.1039/d1tb01024g (PMC8317778; doi:10.1039/d1tb01024g)
Supplement: TB-009-D1TB01024G-s001 [file TB-009-D1TB01024G-s001.pdf]

Electronic Supplementary Material (ESI) for Journal of Materials Chemistry B.
This journal is © The Royal Society of Chemistry 2021

Poly beta amino esters coated emulsions designed for NSAIDs osteoarthritis treatment

by

Tahani Saeedi [1], Polina Prokopovich [1,*]

[1] Cardiff School of Pharmacy and Pharmaceutical Sciences, Cardiff University, UK

Supplementary data

[*] Corresponding author: Dr Polina Prokopovich

School of Pharmacy and Pharmaceutical Sciences

Cardiff University

Redwood Building, King Edward VII Avenue

Cardiff, UK

CF10 3NB

E-mail address: prokopovichp@cf.ac.uk

Table S1. HPLC operating parameters

NSAIDs	Mobile phase	UV detector (nm)
indomethacin	Acetonitrile and acetate buffer pH 3.6 (60:40)	255
ketorolac	Acetonitrile, PBS and acetic acid glacial (26:70:4)	315
naproxen	Acetonitrile and acetate buffer pH 4 (35:65)	230
diclofenac sodium	Acetonitrile and acetate buffer pH 4 (42:58)	276

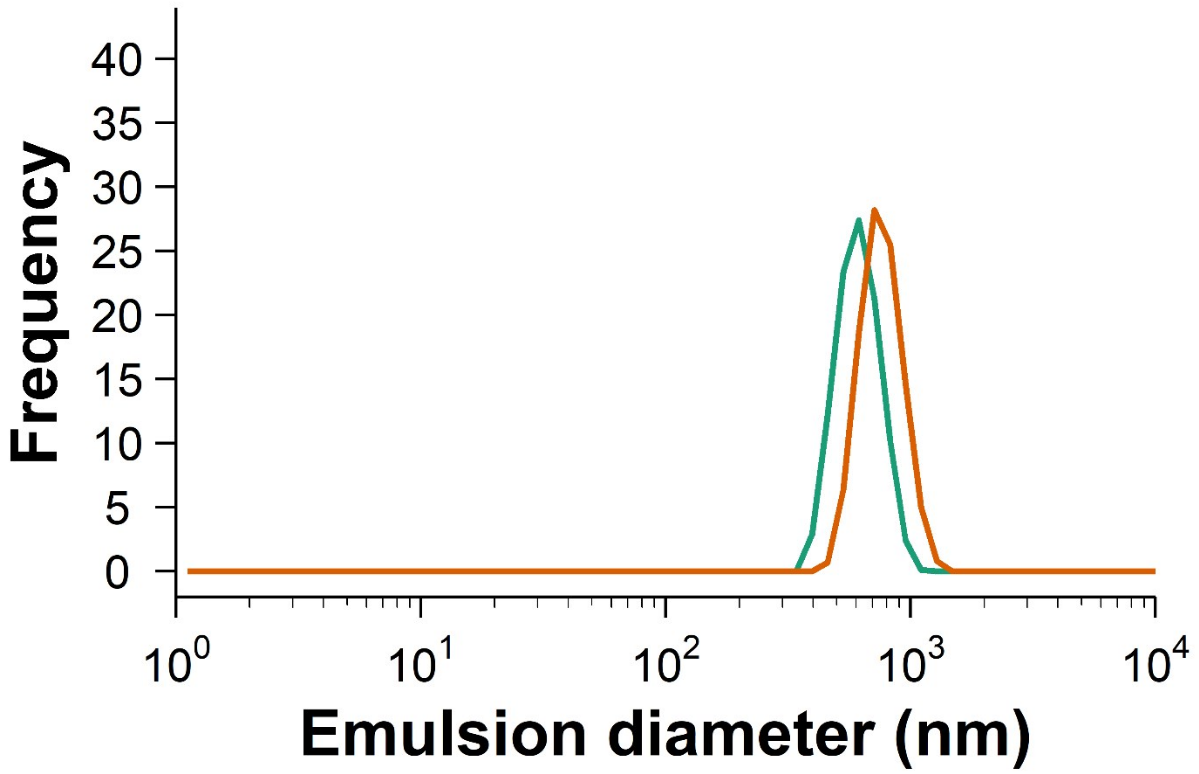

Figure S1. Examples of hydrodynamic size distribution of 10% w/o ( ) uncoated and ( ) PBAE coated pure oleic acid 10% o/w emulsions.

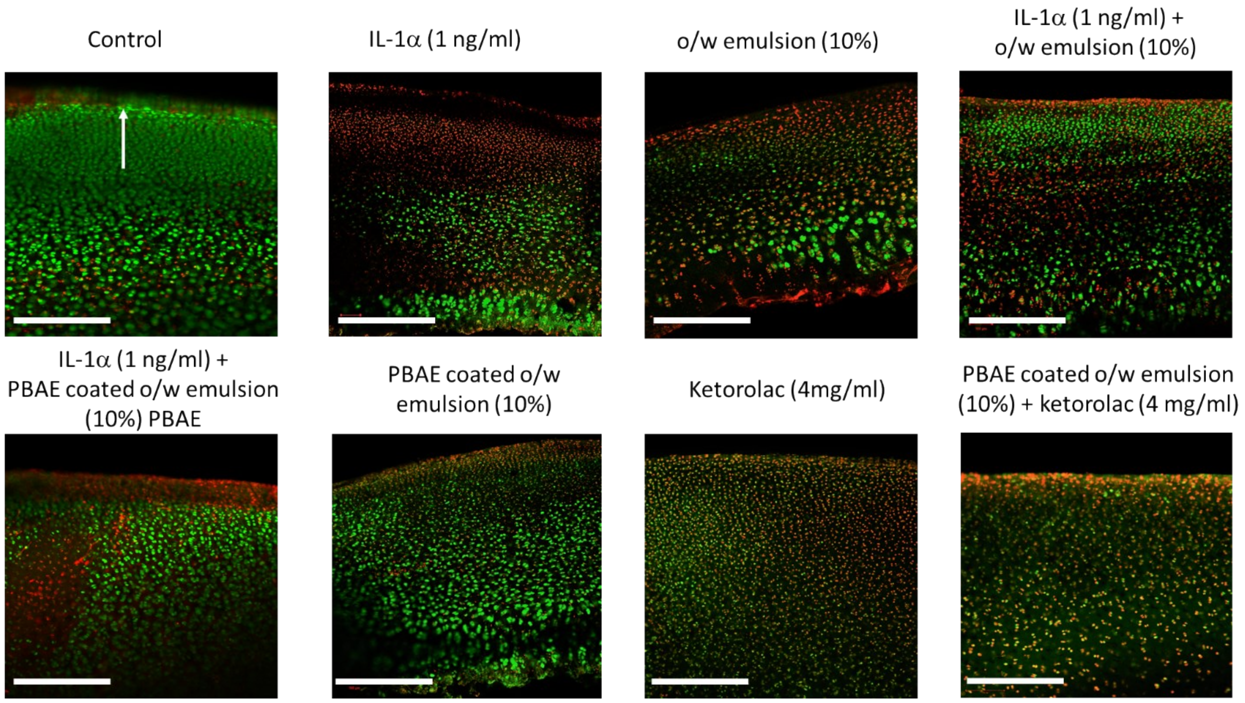

Figure S2. Examples of images of live/dead fluorescently stained bovine explants exposed to IL-1α (1 ng/mL) and ketorolac control samples incubated for 1 day. Scale bar = 500 µm.

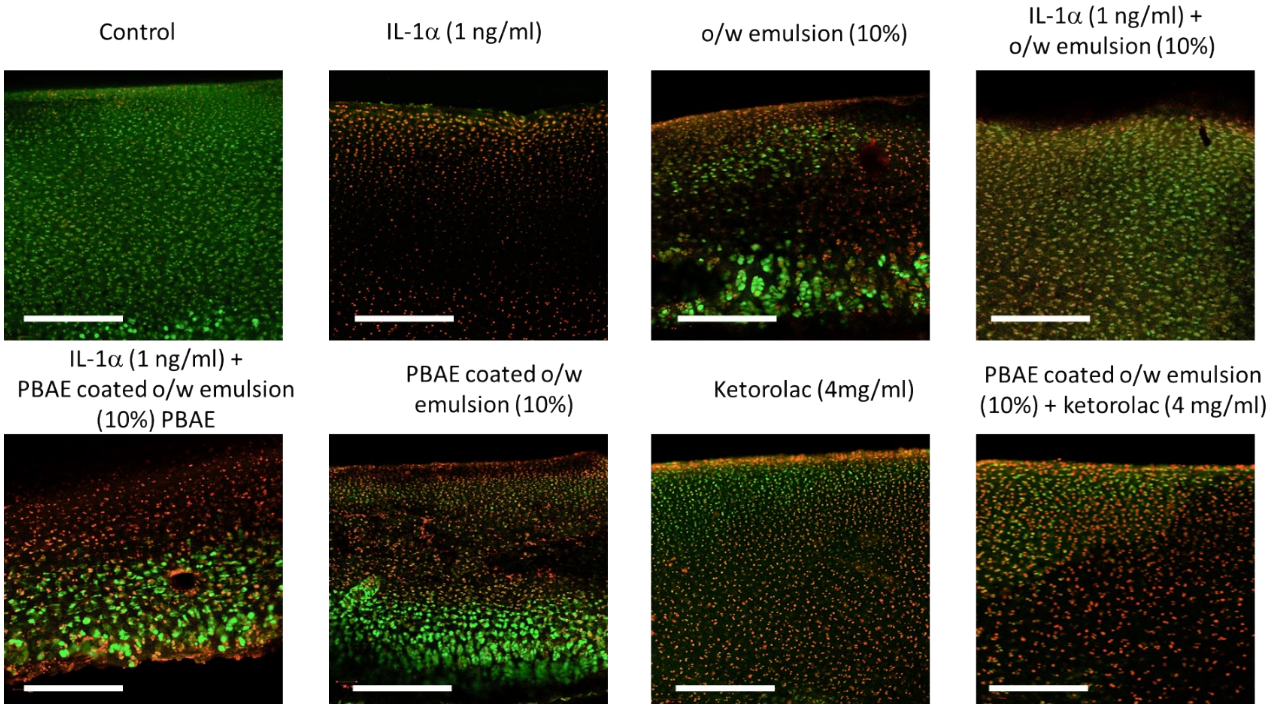

Figure S3. Examples of images of live/dead fluorescently stained bovine explants exposed to IL-1α (1 ng/mL) and ketorolac control samples incubated for 2 days. Scale bar = 500 µm.

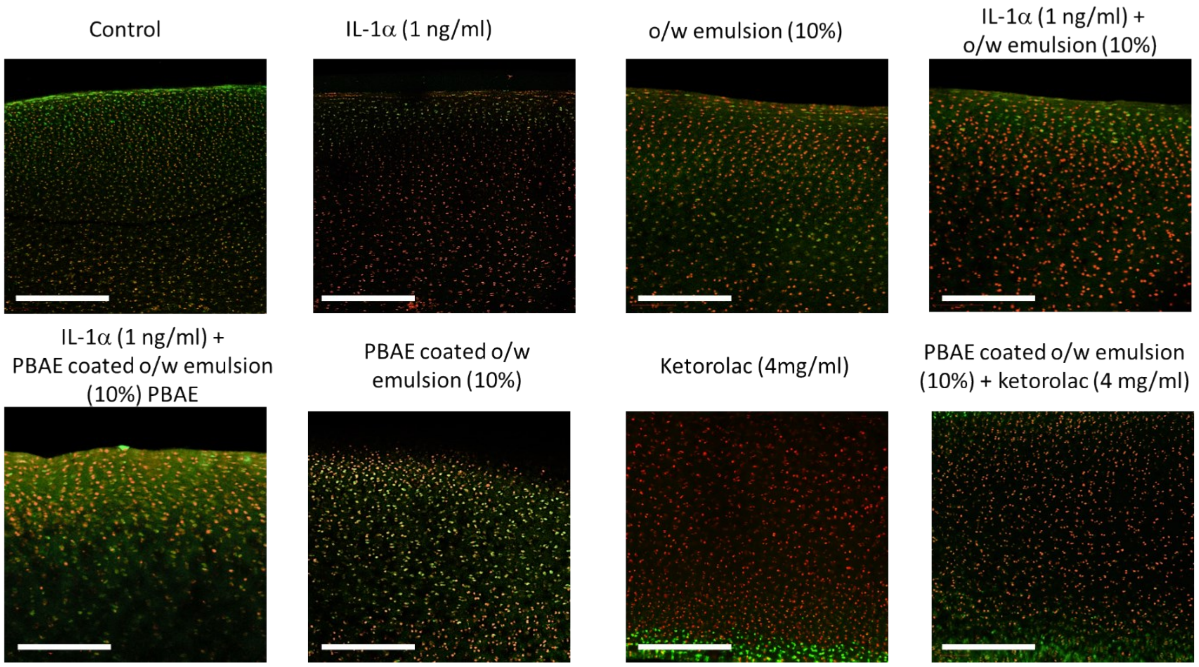

Figure S4. Examples of images of live/dead fluorescently stained bovine explants exposed to IL-1α (1 ng/mL) and ketorolac control samples incubated for 7 days. Scale bar = 500 μm.
